# The impact of traditional mind–body exercises on pulmonary function, exercise capacity, and quality of life in patients with chronic obstructive pulmonary disease: a systematic review and meta-analysis of randomized controlled trials

**DOI:** 10.3389/fmed.2024.1359347

**Published:** 2024-06-19

**Authors:** Mao Sujie, Xiao Kaiwen, Xu Hong, Guo Xiujin

**Affiliations:** ^1^Graduate Department, Harbin Sport University, Harbin, Heilongjiang, China; ^2^School of Sports Industry and Leisure, Nanjing Sport Institute, Nanjing, Jiangsu, China; ^3^Department of Sport and Health, Sangmyung University, Seoul, Republic of Korea

**Keywords:** Qigong, quality of life, chronic obstructive pulmonary disease, pulmonary function, exercise capacity

## Abstract

**Background:**

Chronic Obstructive Pulmonary Disease (COPD) is a chronic condition characterized primarily by airflow obstruction, significantly impacting patients’ quality of life. Traditional mind–body exercises, as a non-pharmacological intervention for COPD, have become a new research focus.

**Objective:**

To assess the impact of traditional mind–body exercises (Tai Chi, Qigong, Yoga) on pulmonary function, exercise capacity, and quality of life in COPD patients. Additionally, to identify the most suitable form of traditional mind–body exercise for different indicators.

**Methods:**

Searches were conducted in databases such as Web of Science, PubMed, EBSCOhost, CNKI, etc., to collect randomized controlled trials (RCTs) evaluating the intervention of traditional mind–body exercises (Tai Chi, Yoga, Qigong) in COPD. The Cochrane evaluation tool was applied for methodological quality assessment of the included literature. Statistical analysis and sensitivity analysis were performed using Revman 5.4 software, while publication bias was assessed using R software.

**Results:**

This study included 23 studies with a total of 1862 participants. Traditional mind–body exercises improved patients’ FEV1% index (WMD = 4.61, 95%CI [2.99, 6.23]), 6-min walk distance (SMD = 0.83, 95%CI [0.55, 1.11]), and reduced patients’ SGRQ score (SMD = −0.79, 95%CI [−1.20, −0.38]) and CAT score (SMD = −0.79, 95%CI [−1.20, −0.38]). Qigong showed the most significant improvement in FEV1% and 6MWT, while Tai Chi primarily improved 6MWT, and the effect of Yoga was not significant. Sensitivity analysis indicated stable and reliable research conclusions.

**Conclusion:**

Traditional mind–body exercises are effective rehabilitation methods for COPD patients, significantly improving pulmonary function, exercise capacity, and quality of life. They are suitable as complementary interventions for standard COPD treatment.

**Systematic review registration:**

[https://www.crd.york.ac.uk/prospero/display-record.php?ID=CRD42023495104], identifier [CRD42023495104].

## 1 Introduction

For Chronic Obstructive Pulmonary Disease (COPD) is a disease characterized by chronic progressive airflow obstruction, partially reversible, and associated with abnormal inflammatory reactions in the lungs to harmful gases or particles such as cigarette smoke (1). Due to the destruction of lung parenchyma in COPD patients, leading to emphysema, chronic inflammation in the lungs, interstitial pneumonia, and reduced lung elastic recoil causing airway narrowing, respiratory airflow restriction occurs, resulting in decreased pulmonary function and breathlessness during physical activity (2). Currently, COPD ranks fourth globally in terms of mortality, following cardiovascular disease, cerebrovascular disease, and HIV/AIDS (3). In China, the prevalence of COPD in the population aged 40 and above is 8.2%, with approximately 32.8 million COPD patients and over one million deaths annually (4). The treatment cost for COPD patients is increasing annually, with average hospitalization expenses ranking among the highest, imposing a heavy economic burden on patients’ families and society (5).

The Global Initiative for Chronic Obstructive Lung Disease (GOLD) advocates various treatment approaches for COPD, including pharmacological treatment, surgical treatment, mechanical ventilation, traditional Chinese medicine, and adjunctive exercise therapy (6). Currently, non-surgical treatments are commonly used in COPD clinical practice, with medications including bronchodilators, corticosteroids, phosphodiesterase inhibitors, expectorants, antioxidants, among others, primarily aimed at controlling chronic inflammation in the lungs of COPD patients (7). While appropriate drug therapy can alleviate symptoms, reduce the frequency of acute exacerbations, improve health status and exercise endurance, no medication has been found to reverse the declining trend in lung function of COPD patients so far (8). Due to the involvement of various pathological changes in COPD, including dysfunction of mucociliary clearance, chronic non-specific inflammation of the airways leading to lung structural damage, and cellular proliferation, drug therapy is challenging (9). GOLD states that non-pharmacological treatments include smoking cessation, physical activity, and pulmonary rehabilitation training, with pulmonary rehabilitation training considered one of the most effective non-pharmacological treatments, effectively alleviating symptoms of dyspnea, improving exercise capacity, and reducing hospitalization rates (8). Pulmonary rehabilitation training helps improve the exercise tolerance of COPD patients, enhance their quality of life, and regular exercise rehabilitation is a core part of pulmonary rehabilitation therapy (10).

Due to the pathological characteristics of COPD patients, they are generally not suitable for moderate to high-intensity physical activity (11). Traditional mind–body exercises, including TC, Yoga (YG), and Qigong (QG), represent a unique traditional fitness and health choice. TC and QG originated from Chinese medicine and martial arts, while YG originated from ancient India as a form of physical exercise (12). These training methods not only share similarities in form but also emphasize body movement, breath control, and relaxation training, each having different benefits for physical rehabilitation (13, 14). Existing studies have confirmed that these traditional mind–body exercises can effectively promote pulmonary rehabilitation and exercise endurance in COPD patients (10). Moreover, traditional mind–body exercises do not require extensive fitness equipment and can be performed in minimal exercise spaces, making them low-cost and easy to learn. These characteristics have played an increasingly important role in the COPD rehabilitation process (15). Therefore, the impact and effectiveness of traditional mind–body exercises on COPD have become a new research focus.

Although numerous randomized controlled trials have explored the effects of traditional mind–body exercises (TC, QG, YG) on COPD, the research results vary due to differences in experimental design, technical methods, sample size, outcome indicators, intervention forms, and periods (16, 17). Additionally, there is no consensus on the specific effects of different forms of exercise on pulmonary function, exercise capacity, and quality of life in COPD patients (18). Furthermore, existing studies are lacking in considering the risk of bias in original studies (19). This study aims to systematically evaluate the improvement effects of these exercises on COPD symptoms through meta-analysis methods, thoroughly analyze their impact on different clinical indicators, address inconsistencies and controversies in existing research, explore the effects of mind–body exercises on improving different indicators in COPD patients, provide effective exercise intervention plans for COPD patients, offer evidence-based medicine for clinical complementary treatments, and promote the development of non-pharmacological treatments for COPD, thereby enhancing the quality of life and health levels of patients.

## 2 Methods

This study employed a systematic review and meta-analysis to comprehensively evaluate the improvement effects of traditional mind–body exercises on COPD patients. The Preferred Reporting Items for Systematic Reviews and Meta-Analyses of individual participant data (PRISMA) checklist (20) was followed to ensure the rigor and transparency of the research (Appendix 1). Additionally, for increased research verifiability, the study was registered on the PROSPERO International Prospective Register of Systematic Reviews (21) platform with registration ID: CRD42023495104.

### 2.1 Inclusion criteria

This study utilized the PICO principle for systematic evaluation, assessing the efficacy of traditional mind–body exercises (TC, Yoga, QG) intervention in patients with COPD. To ensure the inclusiveness of the studies, no restrictions were placed on study types during the literature search. However, during the literature selection process, only randomized controlled trials were chosen.

*Population*: All patients meeting the diagnostic criteria for COPD, regardless of symptomatic presentation.

*Intervention:* Traditional Mind–body exercises. Traditional mind–body exercises are a category of practices that integrate physical workouts with psychological regulation, aiming to improve physical health and enhance mental well-being through mindful and integrated movements. These exercises typically include yoga, TC, and Pilates. The intensity of traditional mind–body exercises generally ranges from low to moderate, making them suitable for people of various ages and physical fitness levels. An effective session of traditional mind–body exercise usually lasts from 30 min to an hour (22, 23).

*Comparison*: Non-mind–body exercise intervention or standard treatment.

*Primary outcome measures*: Forced Expiratory Volume in 1 s percentage (FEV1%), 6-min walk test distance, St. George’s Respiratory Questionnaire (SGRQ) score, and COPD Assessment Test (CAT) score. Inclusion and exclusion criteria followed the PICO framework, ensuring the professionalism and accuracy of the research.

### 2.2 Exclusion criteria

(1) Non-randomized and non-controlled studies are excluded. (2) Case studies or studies focusing on specific cases are excluded. (3) Studies where interventions are simultaneously combined with diet control and other lifestyle changes are excluded. (4) Studies with incomplete or unextractable outcome indicator data are excluded. (5) Review articles are excluded. (6) Animal experiments are excluded. (7) Duplicate publications are excluded. (8) Non-Chinese, non-English, and articles in other languages are excluded.

### 2.3 Literature search strategy

To ensure the comprehensiveness of information sources, this study not only searched English databases such as Web of Science, PubMed, and EBSCO host and the Chinese database CNKI but also reviewed published literature reviews and meta-analyses to discover more relevant references, identifying and including more potential related studies. The search terms were combined with MESH terms and free terms, set according to the PICO principle (Table 1). The search cutoff date was December 31, 2022, aiming to collect randomized controlled trials (RCTs) of traditional mind–body exercises for COPD patients. The search languages were English and Chinese.

**Table 1:** Literature search terms.

Entry	Subject terms	Free terms	Chinese search terms
P	Chronic obstructive pulmonary disease	Chronic Obstructive Lung Disease, Chronic Obstructive Pulmonary Diseases, COPD, Chronic Obstructive Airway Disease, Chronic Obstructive Pulmonary Disease, Airflow Obstruction, Chronic, Airflow Obstructions, Chronic, Chronic Airflow Obstructions, Chronic Airflow Obstruction	慢性阻塞性肺病, 慢阻肺
I	Physical and mental training	Yoga, tai chi, taiji, qigong, ch’i kung, yijinjing, wu qin xi, liu zi jue, six healing sounds, ba duan jin	太极, 太极拳, 气功, 易筋经, 六字诀, 八段锦以及传统养生
C	No exercise group		无运动组
O	Lung function, athletic ability, Quality of Life		肺功能, 运动能力, 生活质量
S	Randomized Controlled Trial [Publication Type], Randomized Controlled Trial		随机对照试验

### 2.4 Study selection

SJM and KWX conducted the initial screening of all retrieved literature, excluding those irrelevant to the research topic based on titles and abstracts. After the initial screening, a detailed reading of the literature was performed to assess whether it met the inclusion criteria. The re-screening process involved a more stringent evaluation, including a full-text reading, assessing study design, sample characteristics, specific content of intervention measures, and quality. In case of discrepancies during the screening or eligibility assessment process, HX made the final decision to ensure the fairness and accuracy of the selection process.

### 2.5 Data extraction

For the literature included after eligibility assessment, data extraction will be conducted, including the first author, publication year, type of study population, sample size, age, intervention measures, use of medications, and outcome indicators. For literature with incomplete data descriptions or lacking key information, authors will be contacted via email to obtain missing data, ensuring the completeness and reliability of the data.

In the data extraction phase, to ensure accuracy, SJM and KWX will independently extract the mean (MEAN) and standard deviation (SD) of all outcome indicators from the included literature. If direct access to MEAN and SD is not possible, appropriate calculation methods will be used to derive MEAN and SD based on the extracted variables. The extracted data results will be cross-checked, and in case of discrepancies or inconsistencies during the data verification process, HX will intervene to make the final decision.

### 2.6 Literature quality assessment

To ensure the quality of the included studies, we will use the Cochrane Collaboration’s “Risk of Bias Assessment” tool for evaluation. This tool addresses aspects such as random allocation, allocation concealment, implementation of blinding, data integrity, selective reporting, and other potential sources of bias. The quality of the studies will be categorized based on the number of criteria met. We will consider the importance and relevance of each domain, and assign weights to the assessment results of each domain to more comprehensively reflect the risk of bias in the studies. Furthermore, we will explore the seven sources of bias in the included studies, clearly identifying areas of bias to better understand the significance of each assessment domain. Through the quality assessment diagram, three color labels—low risk, high risk, and unclear—will be used to indicate the degree of compliance with each criterion, and this will be described and analyzed in the study results.

### 2.7 Statistical analysis

Data extraction and organization for the included studies will be performed using Excel, and Meta-analysis will be conducted using the RevMan 5.4 software package. Continuous data will be represented using the weighted mean difference (WMD) or standardized mean difference (SMD), with a significance level of *α* = 0.05, and both will be presented with a 95% CI. Heterogeneity will be assessed based on the *p*-value from the Q-test combined with *I^2^*. According to the Cochrane Handbook, when the heterogeneity test result *I^2^* ≤ 40%, it is considered that there is low heterogeneity among the included studies. An *I^2^* between 30 and 60% indicates moderate heterogeneity, and an *I^2^* between 50 and 90% suggests significant heterogeneity. When *I^2^* ≥ 75%, it indicates substantial heterogeneity. Therefore, a fixed-effect model is used for meta-analysis when *p* > 0.1 and *I^2^* < 30%; a random-effects model is adopted when heterogeneity test results show *p* ≤ 0.1 or *I*^2^ ≥ 30%.

### 2.8 Other analyses

In this study, subgroup analyses will be conducted to assess the impact of different types of traditional mind–body exercises (TC, YG, QG) on patients with COPD. By comparing the effects of these different intervention forms, a deeper understanding of which types of mind–body exercises are more significantly effective in improving lung function, exercise capacity, and other aspects in COPD patients can be gained. Identifying the specific impact of certain types of exercise interventions on particular patient groups or clinical outcomes and exploring sources of heterogeneity will be the focus.

Through sensitivity analysis, further exploration of the sources of outcome heterogeneity will be undertaken. In this process, each outcome indicator will be included in the analysis, and one by one, exclusions will be made to assess their impact on the combined effect size. If the excluded effect size shows no significant difference compared to the overall effect size, and the point estimate falls within the 95% CI, it indicates good stability of the study results.

This study utilized R software to conduct Egger’s test to evaluate overall publication bias. Egger’s test assesses bias by analyzing the regression relationship between effect size and its standard error, and identifies small sample effects. Small sample studies are prone to random errors, often leading to extreme effect size estimates. Identifying this pattern through Egger’s test allows for the exploration of potential evaluation biases in the included literature, thus enhancing the transparency and reliability of this meta-analysis. For indicators involving more than 10 studies, publication bias analysis was performed by calculating the statistical significance and regression intercepts for different traditional psychophysical exercise groups, and by creating publication bias funnel plots. These plots are color-coded by intervention group to observe symmetry and aid in assessing the presence of publication bias.

## 3 Research results

### 3.1 Literature search results

**Figure 1:**
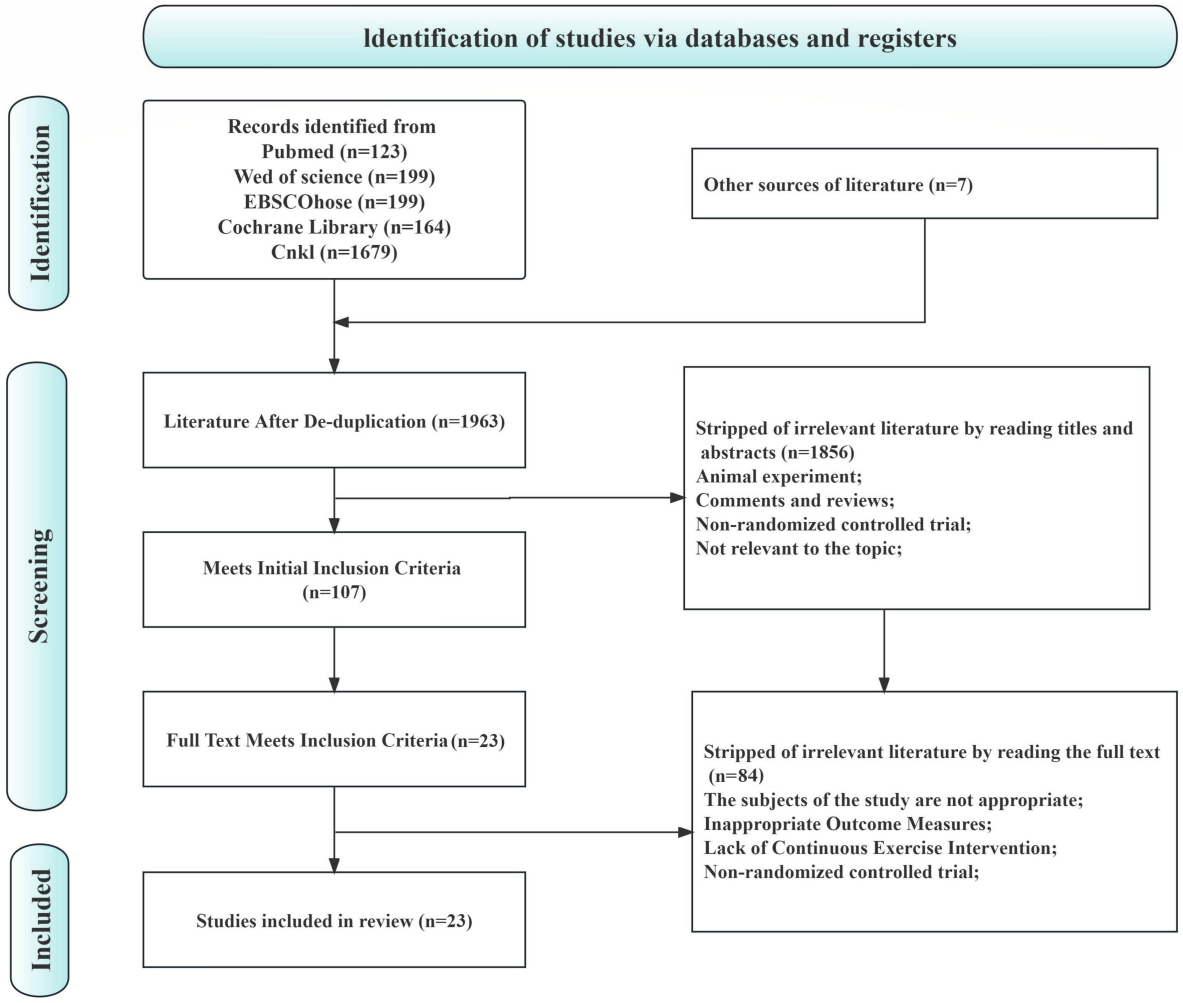
Literature search and screening flow chart.

Through the initial search for relevant articles on the impact of traditional mind–body exercises on COPD, a total of 2,364 literature articles were identified. After removing duplicates using Endnote, 401 articles were excluded. Based on the inclusion and exclusion criteria, 1856 articles were further excluded after reading the titles and abstracts, leaving 107 articles for full-text reading. Finally, a total of 23 articles were included in the study. The flowchart of the literature selection process is shown in Figure 1.

### 3.2 Characteristics of included literature

**Figure 2:**
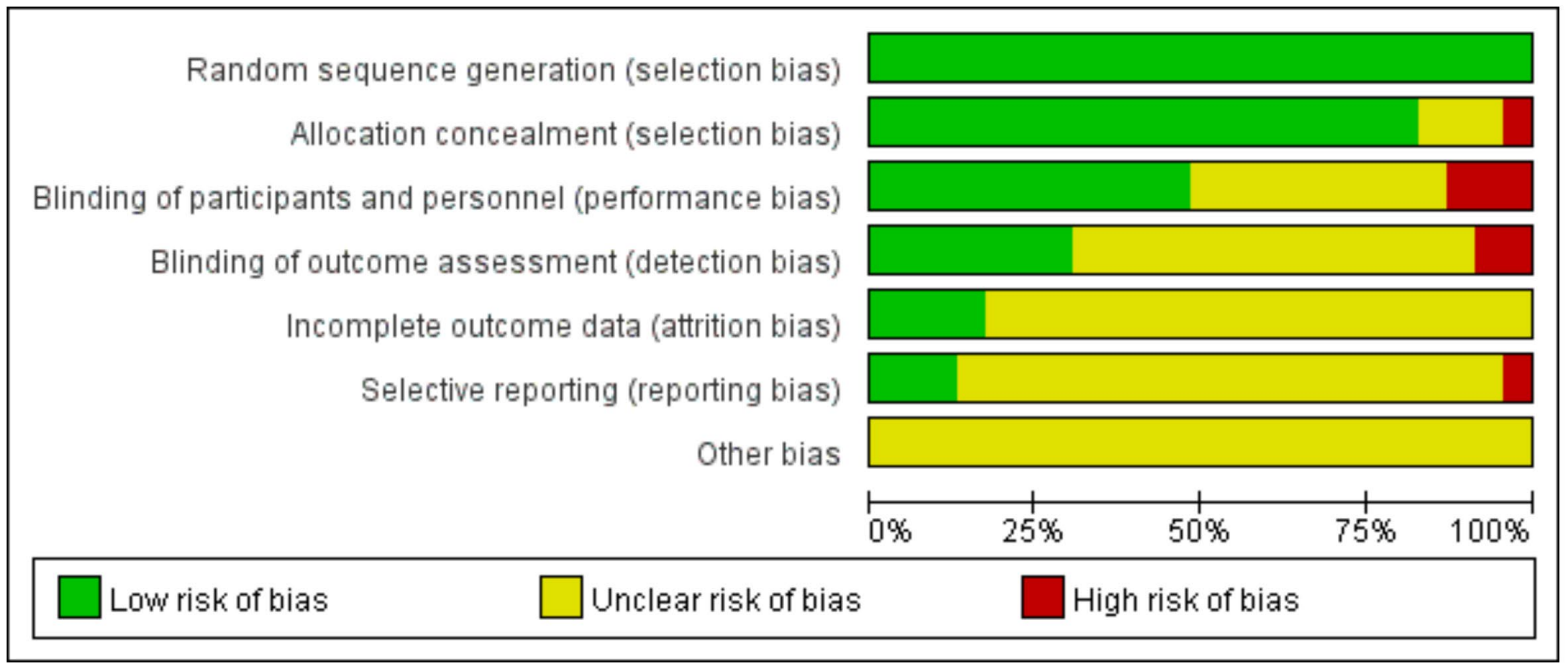
Quality evaluation distribution chart.

This study included 23 articles for Meta-analysis, comprising 1862 participants with an age range from 43 to 78 years. All participants received routine medication treatment. Various mind–body exercise interventions were employed in the studies, including 6 using TC, 4 using YG, and 12 using QG. The interventions varied in duration, frequency, and period, ranging from 8 to 24 weeks and 2 to 10 times per week. The control groups in all included studies did not undergo any form of mind–body exercise intervention, but received routine care, health education, or maintained daily activities. The outcome indicators included a wide range of health-related measurements, such as cardiopulmonary function, physical strength, and psychological health status (Figure 2).

### 3.3 Quality assessment of included literature

The quality of the 23 included articles was assessed using the Cochrane Risk of Bias tool, covering random sequence generation, allocation concealment, blinding (participants, personnel, and outcome assessment), incomplete outcome data, selective reporting, and other potential biases.

This study conducted a quality assessment of 23 articles using the Cochrane bias risk tool, which involved evaluating aspects such as random sequence generation, allocation concealment, blinding of participants, personnel and outcome assessments, incomplete outcome data, selective reporting, and other potential biases. In critical areas like random sequence generation, 21 studies were deemed low risk, indicating robust randomization in their design, which provides a solid foundation for our meta-analysis. In assessing selection bias, it was encouraging to find that 20 studies also displayed low risk, reflecting rigor in participant allocation. However, two studies did not provide sufficient information on allocation concealment, possibly reflecting deficiencies in methodological reporting and transparency in the allocation process. The quality assessment of blinding in outcome evaluations revealed that while 11 studies implemented strict blinding measures and were considered low risk, two studies had evident issues with blinding during outcome assessment, and 14 studies did not report details on the implementation of blinding for outcome evaluations thoroughly. To assess the impact of these issues on the overall conclusions, sensitivity analyses were performed, excluding studies one by one to ensure the robustness of the meta-analysis results. Regarding attrition bias, only two studies explicitly reported the details of participant dropouts, one study reported dropouts without specifying the reasons, and 19 studies lacked descriptions of dropouts. Sensitivity analyses will be conducted for these studies to fully understand and interpret the impact of missing data on the study outcomes. When evaluating whether the included studies were prone to selective reporting, it was found that most studies (18) did not explicitly state this risk. Only three studies were judged to have a low risk of selective reporting, and one study was considered high risk. This suggests that the results of these studies need to be interpreted with caution. In assessing other potential biases, a systematic evaluation was conducted, and it was concluded that the included studies did not exhibit significant other biases.

**Figure 3:**
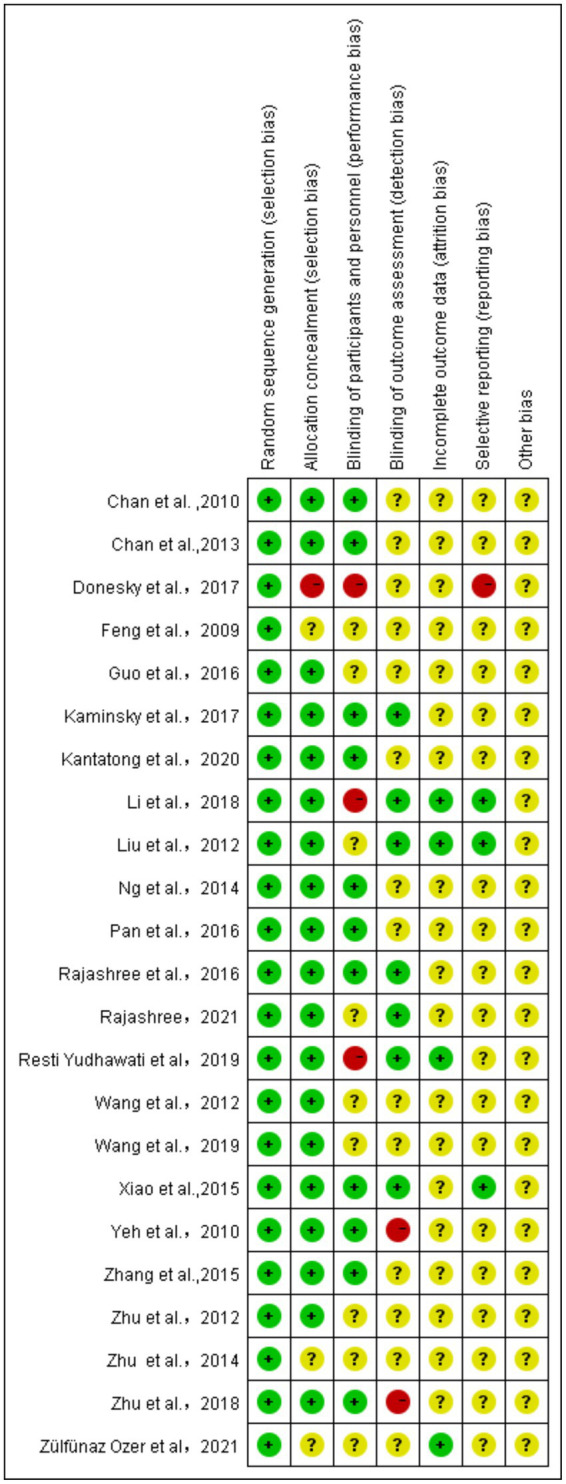
Quality evaluation summary chart.

This meta-analysis followed a high standard quality assessment process, providing solid evidence for non-pharmacological treatments for patients with COPD. Although the included studies demonstrated commendable rigor in randomization, uncertainties in blinding and reporting transparency remain (Figure 3). The 23 included articles were marked as high risk or unclear in several areas, necessitating a cautious evaluation of the credibility of their conclusions. Future research should continue to enhance reporting quality to further optimize the methodology of systematic reviews and provide more precise bases for clinical decision-making.

### 3.4 Meta-analysis results

#### 3.4.1 Effect of traditional mind–body exercises on FEV1% in COPD patients

This study included a total of 23 articles for the evaluation of the intervention effects of traditional mind–body exercises on FEV1% in patients with COPD. The literature was classified based on different forms of traditional mind–body exercises, including TC with 5 studies, QG with 15 studies, and YG with 4 studies.

**Figure 4:**
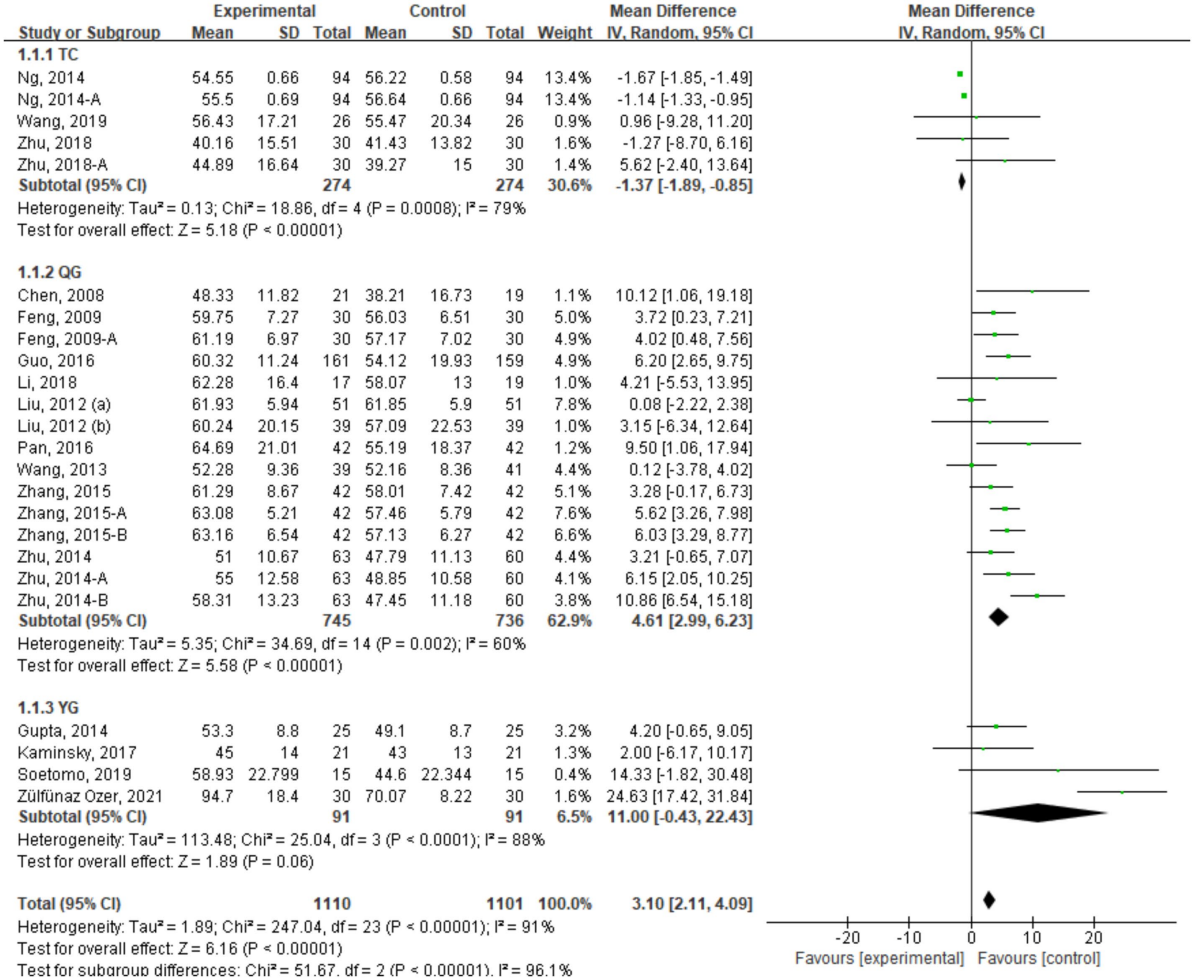
FEV1% indicator combined effect size forest plot.

Figure 4 presents the meta-analysis results of the combined effect size data after intervention on FEV1%. It can be observed that traditional mind–body exercises significantly improved the FEV1% indicator in COPD patients (WMD = 2.54, 95% CI [1.61, 3.47]). Subgroup analysis based on different exercise forms revealed that TC group had a combined effect size on FEV1% of WMD = −1.37, 95% CI [−1.89, 0.85], YG group had an WMD of 4.30, 95% CI [2.99, 6.23], and Qigong group had a combined WMD of 4.61, 95% CI [2.99, 6.23]. The results indicate that compared to the control group, TC did not improve lung function in COPD patients, while YG and Qigong could enhance FEV1% in COPD patients, with Qigong showing the most significant improvement.

#### 3.4.2 Effect of traditional mind–body exercises on exercise capacity

**Figure 5:**
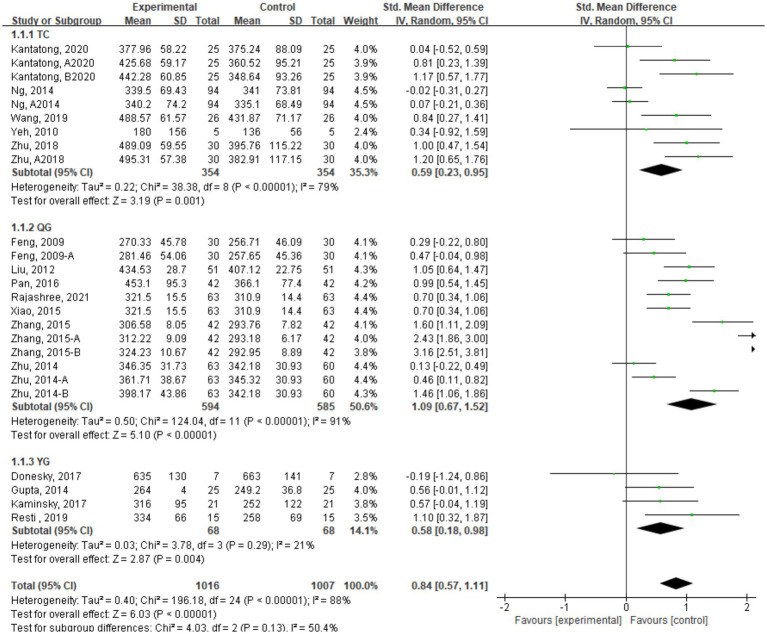
6MWT indicator combined effect size forest plot.

This study included a total of 23 studies with 1993 COPD patients. The studies were categorized into three groups based on different forms of traditional mind–body exercises: TC group with 9 studies, Qigong group with 12 studies, and YG group with 4 studies. Figure 5 shows the meta-analysis results of the TMB group compared to the control group (Table 2).

**Table 2:** Basic characteristics of included studies.

Researchers, year	Medication status	Population characteristics		Intervention group		Control group	Outcome measures
		n	Age/Years	Form of exercise	Duration (Time × Frequency × Duration)		
Chan et al., 2010	Prescription drug	206	54.5 ± 10.75	TC	60 min/time×2 times/week×12 weeks	Usual care	2, 3
Chan et al., 2013	Prescription drug	206	54.5 ± 10.75	TC	60 min/time×2 times/week×12 weeks	Usual care	2, 3
Donesky et al., 2009	Conventional drug	29	69.9 ± 9.5	YG	60 min/time×2 times/week×12 weeks	Usual care	2
Donesky et al., 2017	Conventional drug	15	73 ± 14.3	YG	60 min/time×2 times/week×8 weeks	Health education	1
Kaminsky et al., 2017	Standard drug	43	68 ± 7	YG	30 min/time×2 times/week×12 weeks	Individual education	1, 2
Kantatong et al., 2020	Standard drug	50	69.68 ± 7.67	TC	3 times/week×24 weeks	Usual care	1, 2, 3
Li et al., 2018	Standard drug	40	66 ± 9	QG	60 min/time×6 times/week×24 weeks	No exercise	1
Liu et al., 2012	Conventional drug	118	61.82 ± 7.69	QG	60 min/time×3 times/week×24 weeks	Conventional drug treatment	1, 2
Ng et al., 2014	Conventional drug	192	74.13 ± 6.81	QG	60 min/time×2 times/week, 6 weeks	Pulmonary rehabilitation program	1, 2, 3
Rajashree et al., 2016	Conventional drug	72	53.69 ± 5.66	YG	90 min/time×6 times/week×12 weeks	Usual care	1, 2, 4
Rajashree, 2021	Conventional drug	20	65.50 ± 6.26	QG	30 min/time× times/week×12 weeks	Bicycle strength training	3, 4
Wang et al., 2019	Conventional drug	20	67.83 ± 5.32	TC	times/week×3 months	Daily activities	1, 2, 4
Xiao et al., 2015	Conventional drug	126	70.9 ± 1.4	QG	45 min/time× times/week×24 weeks	Walk	1, 2
Yeh et al., 2010	Conventional drug	92	68 ± 8	TC	60 min/time×2 times/week×24 weeks	Health education	2
Zhang et al., 2015	Conventional drug	100	64.77 ± 11.07	QG	60 min/time×1 times/week×24 weeks	Usual care	1, 2, 4
Zhu et al., 2018	Conventional drug	60	67.87 ± 5.22	TC	45 min/time×3 times/week×12 weeks	No exercise	1, 2
Feng et al., 2009	Conventional drug	60	62 ± 5	QG	45 min/time×10 times/week×24 weeks	Conventional drug treatment	2
Guo et al., 2016	Conventional drug	161	64.15 ± 8.9	QG	30 min/time×4 times/week×24 weeks	Conventional drug treatment	1
Pan et al., 2016	Conventional treatment	84	61. 8 ± 7. 2	QG	30 min/time×2 times/week×24 weeks	Usual care	2
Wang et al., 2012	Conventional drug	63	73.52 ± 1.2	QG	30 min/time×2 times/week×24 weeks	Usual care	4
Zhu et al., 2012	Conventional treatment	45	60 ± 5	QG	7 times/week×12 weeks	Baseline treatment	4
Zhu et al., 2014	Conventional drug	60	68 ± 9.2	QG	30 min/time×14 times/week×24 weeks	Conventional drug treatment	1, 2
Resti Yudhawati et al., 2019	Conventional drug	30	64.40 ± 10.453	YG	60 min/次 × 2times/week×12 weeks	Pulmonary rehabilitation training	1, 2, 3
Zülfünaz Ozer et al., 2021	Conventional drug	60	64.85 ± 9.26	YG	60-90 min/次 × 2times/week×8 weeks	No Exercise Intervention	1

n, sample size; TC, Tai Chi exercise; YG, Yoga exercise; QG, Qigong exercise; 1, FEV1%; 2, 6MWT; 3, SGRQ score; 4, CAT score.

The 6-min walk test (6MWT) is a precise indicator to measure the exercise endurance of COPD patients, and its results are positively correlated with exercise capacity. The meta-analysis results of the combined effect size for the included studies showed that traditional mind–body exercises could improve the 6MWT indicator in COPD patients (SMD = 0.83, 95% CI [0.55, 1.11]), with statistical significance.6MWT.

Subgroup analysis of different forms of traditional mind–body exercises revealed that the Qigong group had the most significant improvement, with a combined effect size of SMD = 1.09, 95% CI [0.67, 1.52]. The TC group showed an effect size of SMD = 0.59, 95% CI [0.23, 0.95], followed by the YG group with a combined effect size of SMD = 0.46, 95% CI [0.07, 0.85].

#### 3.4.3 Effect of traditional mind–body exercises on SGRQ scores

**Figure 6:**
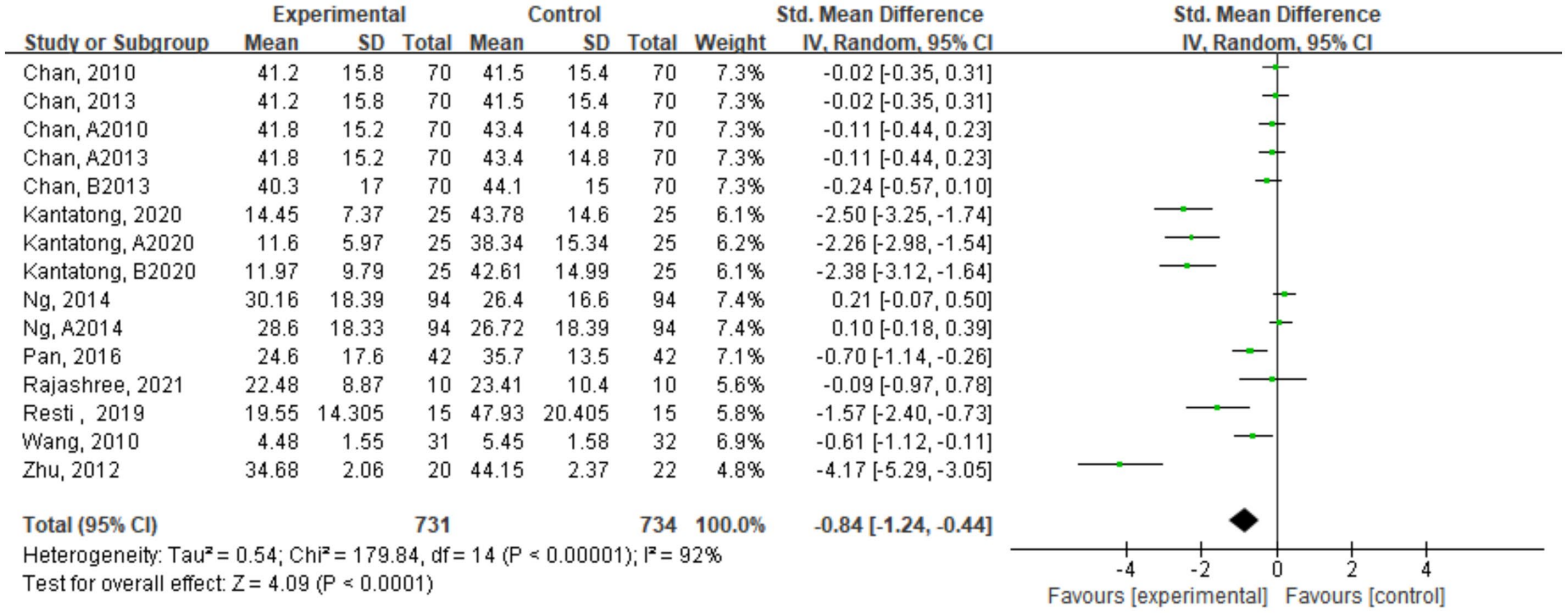
Forest plot of combined effect sizes of SGRQ score indicators.

A total of 14 studies with 1,435 COPD patients were included in the analysis of the impact of traditional mind–body exercises on SGRQ scores. Figure 6 shows the meta-analysis results of the TMB group compared to the control group for SGRQ scores, and due to high heterogeneity, a random-effects model was selected.

Quality of life scores were assessed using the SGRQ to evaluate the quality of life of COPD patients. The scores are negatively correlated with the quality of life of COPD patients. The meta-analysis results indicated that traditional mind–body exercises could improve SGRQ scores in COPD patients (SMD = −0.79, 95% CI [−1.20, −0.38]), suggesting that compared to the control group, traditional mind–body exercises could enhance the quality of life of COPD patients.

#### 3.4.4 Effect of traditional mind–body exercises on CAT scores

**Figure 7:**
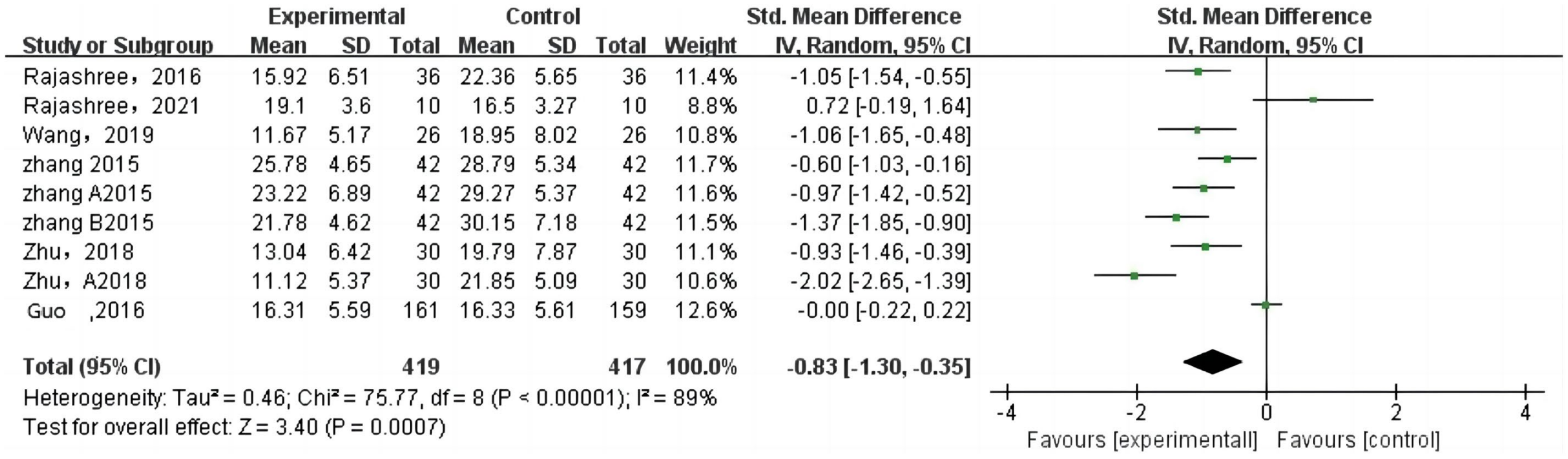
Forest plot of combined effect sizes of CAT score indicators.

The CAT is used to assess and test symptoms in COPD patients, and its test scores are positively correlated with COPD symptoms. This study included a total of 9 studies with 836 COPD patients. Figure 7 presents the meta-analysis results of CAT score effect sizes. The results showed that traditional mind–body exercises could reduce CAT scores in COPD patients, with a combined effect size of SMD = -0.79, 95% CI [−1.20, −0.38], *p* < 0.05, indicating statistical significance. This suggests that compared to the control group, traditional mind–body exercises could decrease CAT scores in COPD patients.

#### 3.4.5 Publication bias analysis

To assess publication bias within groups, the number of studies in each subgroup was less than 10, leading to the setting of a threshold (*k*.min) at 3 in R to conduct Egger’s test. Table 3 shows that the *p*-values for the TC, QG, and YG groups under the FEV1% indicator are all above 0.5, indicating no significant publication bias in these subgroups. High Tau^2^ values, particularly in the YG group, indicated notable small-sample effects impacting the reliability of the meta-analysis. For the 6MWT indicator, the TC group’s *p*-value of 0.423 and a Tau^2^ of 47.3122 suggested moderate variability among study effect estimates with no significant publication bias. The QG group had a *p*-value close to significance at 0.0626, suggesting potential publication bias, and a high Tau^2^ of 98.304 indicating substantial heterogeneity. The YG group had a low Tau^2^ of 6.3086 and a stable bias estimate despite a *p*-value of 0.0822, reflecting characteristics of small-sample studies.

**Table 3:** Summary of publication bias analysis results.

Item	Subgroup	*t* value	Df	*p*-value	Bias estimate	SE of bias estimate	Tau^2^
FEV1%	TC	−0.74	3	0.5123	−2.6684	3.6003	37.1262
	QG	−1.61	13	0.1313	−3.1008	1.9254	8.7897
	YG	0.52	2	0.6538	5.3435	10.2374	63.5282
6WMT	TC	0.85	7	0.423	5.5922	6.5727	47.3122
	QG	2.1	10	0.0626	10.6635	5.0897	98.304
	YG	3.27	2	0.0822	4.914	1.5035	6.3086
SGRQ	–	2.08	13	0.0575	11.9381	5.7298	–

**Figure 8:**
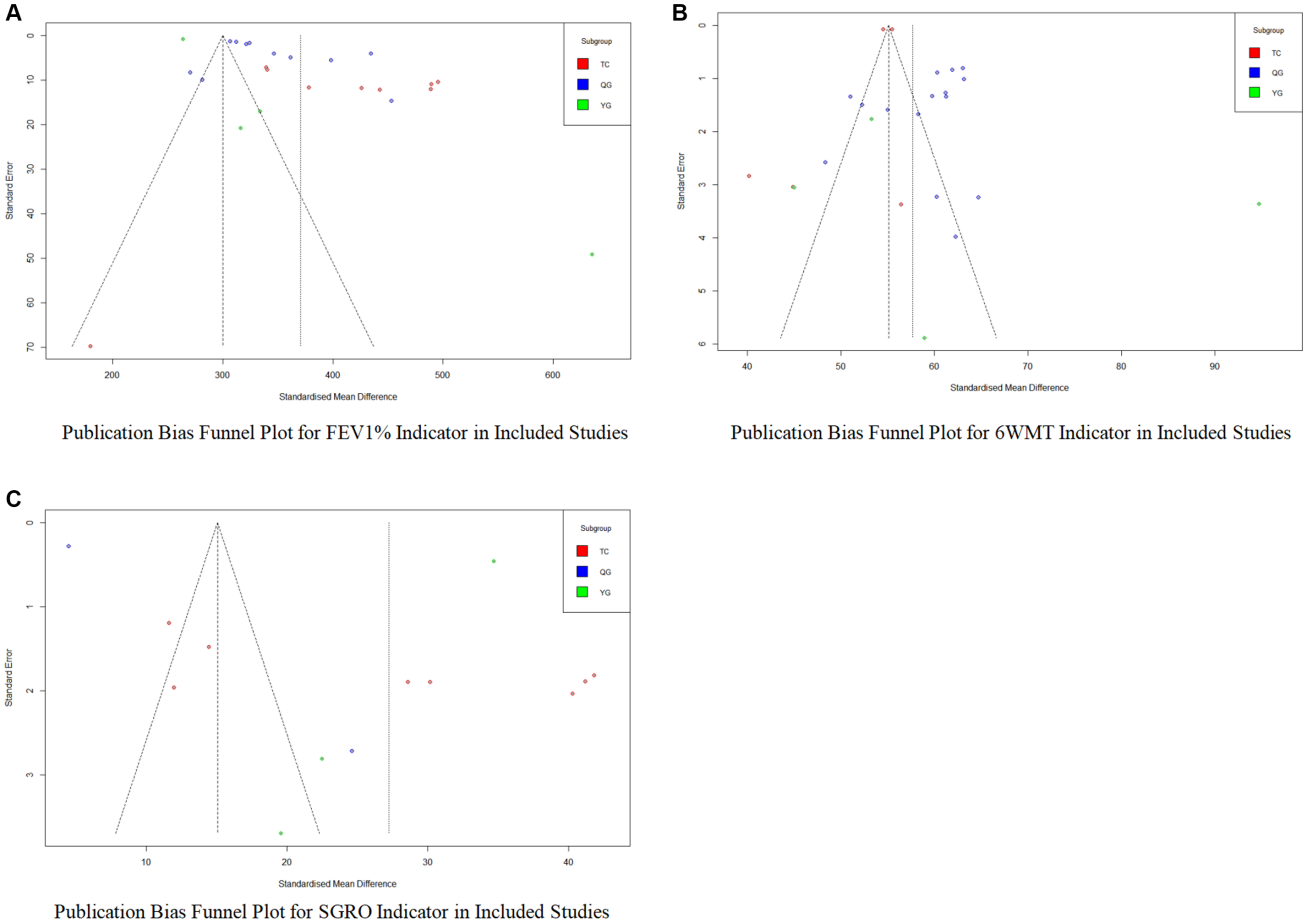
Publication bias analysis funnel plot. **(A)** Publication bias funnel plot for FEV1% indicator in included studies; **(B)** Publication bias funnel plot for 6MWT indicator in included studies; **(C)** Publication bias funnel plot for SGRQ indicator in included studies.

For the SGRQ indicator, due to the limited number of included studies, no subgroup analysis was performed. The *p*-value of 0.0575 did not confirm significant publication bias, but the regression intercept of 11.9381 suggested a potential high bias, and a Tau^2^ of 252.344 indicated significant variability among study effect estimates, potentially influenced by small-sample effects. Funnel plots (Figure 8) showed symmetrical distributions for FEV1% and 6MWT, suggesting insignificant publication bias, while asymmetry in SGRQ suggested potential bias.

No publication bias analysis was conducted for the CAT indicator due to an insufficient number of studies.

#### 3.4.6 Sensitivity analysis

To further identify potential sources of heterogeneity, sensitivity analysis was conducted in this study. This analysis considered all outcome indicators and systematically excluded each study to observe its impact on the overall combined effect size. The results indicated that even when excluding any single study, there was no statistically significant difference observed between the combined effect size of the remaining studies and the overall effect size. The point estimates after each exclusion consistently fell within the 95% CI range of the overall effect size. This finding suggests that the influence of individual studies on the overall analysis results is limited, and the conclusions of this study are not overly influenced by any single study. Therefore, the results of this study are considered to have good stability.

## 4 Discussion

As an integral part of the comprehensive management of COPD, exercise rehabilitation is increasingly widely used in clinical treatment (61). This study conducted a meta-analysis of 23 studies, systematically evaluating the impact of traditional mind–body exercises on the pulmonary function, exercise capacity, and quality of life of COPD patients from the perspective of evidence-based medicine. Pulmonary function was assessed through direct measurement of FEV1%, while exercise capacity and quality of life were measured using indirect indicators such as the 6MWT, CAT scores, and St. George’s Respiratory Questionnaire (SGRQ). The results of the analysis show that traditional mind–body exercises, especially QG, significantly improve the pulmonary function of COPD patients, while TC, QG, YG, and other exercises generally enhance the patients’ exercise capacity. Additionally, YG and QG exercises have shown positive effects on improving the quality of life for COPD patients.

### 4.1 Impact of traditional mind–body exercises on pulmonary function

COPD is characterized by airflow limitation (9), leading to clinical syndromes such as difficulty breathing after physical activity. The reduction in physical activity among patients contributes to the dysfunction of body functions (24), forming a vicious cycle. Studies have shown an increase in CD8+ T cells in the peripheral airways of COPD patients, with the inflammation of airways inversely proportional to FEV1 airflow limitation. The CD4+ /CD8 + ratio in the bronchial glands of COPD patients is positively correlated with FEV1/FVC, reflecting airflow limitation in pulmonary function testing as a decrease in FEV1% and presenting varying degrees of pulmonary function levels and symptoms of respiratory difficulty (25).

The meta-analysis in this study demonstrates that traditional mind–body exercises can improve FEV1% in COPD patients. However, among different forms of traditional mind–body exercises, TC does not show improvement in FEV1% for COPD patients. This is consistent with the conclusion reached by Ng et al. in a randomized controlled trial, where TC improved physiological functions and exercise capacity in COPD patients, but the improvement in pulmonary function was not statistically significant (26), aligning with previous extensive research results (27, 28).

When COPD patients practice QG, the combination of body and breath generates different internal pressures in the abdomen, guiding the flow of qi and blood along various organs to regulate organ functions, thereby improving the lungs (9). QG practitioners, through abdominal breathing, can improve abnormal breathing patterns (29), increase tidal volume, enhance alveolar ventilation, reduce respiratory energy consumption, and alleviate symptoms of respiratory difficulty (30). At the same time, QG exercises can enhance the body’s immune system, increasing CD4+ indicators in the peripheral blood (31), thereby improving FEV1%, reducing airway inflammation, and ameliorating symptoms of airflow obstruction in COPD patients. YG, when practiced by COPD patients, can increase tidal volume and forced vital capacity, decrease respiratory rate, and improve pulmonary function (32). Additionally, YG can enhance the coordination of muscles, joints, and the entire musculoskeletal system, strengthening muscles in the back, chest, and abdomen, increasing breathing depth, and improving pulmonary function (33). Furthermore, during YG practice, respiratory endurance can be increased through breathing exercises, leading to a greater intake of oxygen, improving lung capacity, and increasing overall vitality and endurance (34).

### 4.2 Impact of traditional mind–body exercises on exercise capacity

A decline in exercise capacity is one of the main symptoms of COPD and a determining factor leading to a decrease in patient quality of life and an increase in mortality rates (35). Due to airway remodeling and obstructive emphysema in COPD patients, pulmonary function decreases, causing respiratory difficulties and subsequent reductions in exercise capacity (36). Additionally, incomplete reversible airflow limitation, ventilatory dysfunction, alterations in glucose metabolism pathways, and increased anaerobic glycolysis with lactic acid accumulation contribute to the proliferation of type II muscle fibers and abnormalities in skeletal muscle structure and function, resulting in decreased exercise capacity (36, 37). The 6MWT is commonly used to assess exercise capacity in COPD patients (38). Furthermore, the 6MWT is correlated with patient pulmonary function, reflecting changes in lung function. Due to its simplicity, convenience, and practicality, it serves as a long-term monitoring tool (39).

Compared to the control group, the TC group showed an improvement in 6MWT distance. During TC practice, the close coordination of muscle activity and breathing under the regulation of the cerebral cortex’s respiratory control center increases chest expansion and contraction, significantly changing intrathoracic pressure, enhancing alveolar ventilation, and reducing excessive airway resistance in COPD patients, ultimately improving exercise capacity (40). TC, as a form of low-to-moderate-intensity aerobic exercise, can enhance muscle oxygen metabolism (41). A randomized controlled trial by Du demonstrated that after 12 weeks of TC compared to conventional exercise and no exercise intervention groups, the exercise capacity of COPD patients in the TC group was significantly higher. This suggests that TC can improve pulmonary function, blood oxygen saturation, exercise capacity, and quality of life in COPD patients (42, 43).

Among the three traditional mind–body exercises, the QG group showed the most significant improvement in 6MWT for COPD patients. QG, through specific breathing techniques, controls the rise and fall of internal breath, coordinates with QG movements, regulates visceral organs, and enhances fitness effects. This leads to improved pulmonary function and increased exercise capacity for COPD patients (44). From a traditional Chinese medicine perspective, COPD pathology often progresses from Qi deficiency and Yin deficiency to Yin deficiency and Yang deficiency, causing “lung swelling” and severely affecting patients’ labor capacity and quality of life. QG, by controlling inhalation and exhalation, acts to adjust the spirit, breath, and body, thus improving the pathological mechanism of COPD, alleviating respiratory difficulties, and enhancing exercise capacity (41). Additionally, due to reduced physical activity, decreased exercise tolerance, muscle function impairment, and reduced muscle strength and endurance in COPD patients (45), QG can reduce respiratory load, stimulate the sympathetic nervous system, improve oxygen transport function in the blood, increase muscle endurance, and improve muscle function (46), thereby enhancing exercise capacity.

### 4.3 Impact of traditional mind–body exercises on quality of life

COPD patients often experience a decline in quality of life, and routine biological indicators cannot comprehensively reflect patients’ health status. Assessing the quality of life in patients not only reflects their physical function but also evaluates their psychological state and social adaptability (47). The SGRQ is a commonly used quality of life assessment tool for COPD patients, focusing on symptoms, activities, and psychology. Quality of life reflects the comprehensive impact of the disease and treatment measures on patients’ physical function, psychological status, and social adaptation. Changes in quality of life are influenced by various factors, but as COPD patients’ conditions improve, their quality of life also improves. According to the Global Initiative for Chronic Obstructive Lung Disease (GOLD) recommendations, the CAT can be used as an evaluation method for breathlessness in COPD patients (48). This scale tests COPD patients on items such as sputum, cough, sensations during stair climbing or uphill walking, chest tightness, confidence, household activities, energy, and sleep. It provides an assessment of patients’ daily lives and physical and mental health, serving as an independent and comprehensive evaluation tool (49). Both the SGRQ and CAT scales are effective tools for assessing the health-related quality of life of stable COPD patients.

Traditional mind–body exercises can improve the quality of life of COPD patients for three main reasons: (1) The most fundamental pathophysiological change in COPD is obstructive ventilatory impairment, making pulmonary function a crucial factor influencing patient quality of life. Through traditional mind–body exercises and respiratory regulation, patients increase thoracic and lung activity, expand chest volume, promote alveolar expansion, improve respiratory muscle fatigue, and enhance pulmonary function, thus alleviating respiratory difficulties and improving quality of life (50, 51). (2) Long-term engagement in traditional mind–body exercises significantly improves tension, anxiety, anger, hostility, fatigue, and other emotions in patients (52). Traditional mind–body exercises improve the psychological well-being of COPD patients, leading to enhanced quality of life. (3) Simultaneously, during traditional mind–body exercises, many movements require mutual cooperation, promoting interpersonal interactions. This relieves patient self-isolation, alleviates feelings of emptiness and loneliness, adjusts negative emotions, and integrates patients into social groups. The social function improvement through traditional mind–body exercises contributes to an enhanced quality of life (53, 54).

In our study, the SGRQ indicator demonstrated significant publication bias which may have impacted the overall results, whereas the FEV1% and 6MWT indicators did not show significant publication bias. The Egger’s test for the YG group indicated the presence of small-sample effects, evidenced by high SE and Tau^2^ values. Similarly, the 6MWT’s TC group displayed small-sample impacts. However, sensitivity analyses and the leave-one-out method showed that these small samples did not significantly affect the study outcomes. Despite meta-analyses indicating that traditional psychophysical exercises positively affect lung function, YG did not show significant effects, contradicting previous findings (55, 56). YG can alleviate breathing difficulties and enhance exercise capacity in COPD patients, improving symptoms and reducing pain (57). The primary reasons for the nonsignificant improvement in lung function from YG interventions may be the small-sample effects and insufficient statistical power, which could lead to underdetection of small but meaningful benefits. These effects also cause wider 95% CI, increasing uncertainty in result interpretation (58). Inconsistencies in intervention implementation and publication bias toward positive results could further skew meta-analysis outcomes toward optimism. High heterogeneity in study results might be due to variations in COPD severity, age, and comorbidities among participants. Future research should address these issues with larger sample sizes, more standardized implementation, and stricter study designs. TC has shown consistent improvements in the exercise capacity of COPD patients, aligning with past research (59, 60), indicating that despite small-sample effects, the results are consistent with historical data.

### 4.4 Limitations and shortcomings of the study

1. This study only included publicly published literature, excluding unpublished literature, which may affect the comprehensiveness of the research.
2. Due to the limited number of studies included, subgroup analyses for SGRQ and CAT scores were not conducted, making it impossible to determine which form of psychophysical exercise is most effective for intervening in COPD patients.
3. The included literature did not discuss the form of pharmacological treatment (types of drugs, duration of drug use), which could introduce bias due to differences in drug use. And some included literature did not provide relevant outcome indicator data.
4. Due to the limited number of studies included, no publication bias analysis was conducted for the CAT score, and thus it is not possible to ascertain the presence of bias.
5. One limitation of this study is the impact of small-sample effects, particularly in the FEV1% and 6MWT subgroup analyses, where the limited number of studies may result in insufficient statistical power, thereby affecting the interpretation and reliability of the results. Future research should consider increasing the sample size and employing stricter RCTs designs to enhance the stability and generalizability of the findings.

## 5 Conclusion

The results of this meta-analysis indicate that traditional mind–body exercises have a significant improvement effect on lung function, exercise capacity, and quality of life in patients with COPD. Regarding the improvement of the pulmonary function indicator FEV1%, QG demonstrated the most significant effect, while TC did not show a corresponding improvement in this indicator. In terms of exercise capacity, QG, TC, and YG all contributed to an increase in exercise endurance for COPD patients. The evaluation of the improvement effect on quality of life confirmed that traditional mind–body exercises can effectively enhance the quality of life of COPD patients. Through sensitivity analysis, the conclusions of this study demonstrate good stability and reliability.

This study emphasizes the significant value of traditional mind–body exercises in the comprehensive rehabilitation strategy for COPD, revealing their remarkable benefits in promoting lung function recovery, enhancing exercise capacity, and improving quality of life. These results provide valuable guidance for clinical complementary therapy, recommending the inclusion of traditional mind–body exercises in the standard treatment plan for COPD patients as an important adjunctive treatment method.

## Data availability statement

The original contributions presented in the study are included in the article/Supplementary material, further inquiries can be directed to the corresponding author.

## Author contributions

MS: Data curation, Methodology, Software, Visualization, Writing – original draft, Writing – review & editing. XK: Conceptualization, Data curation, Writing – review & editing. XH: Writing – original draft, Data curation, Methodology, Software. GX: Writing – original draft, Writing – review & editing, Data curation, Investigation, Software.
